# CLEC3A, MMP7, and LCN2 as novel markers for predicting recurrence in resected G1 and G2 pancreatic neuroendocrine tumors

**DOI:** 10.1002/cam4.2232

**Published:** 2019-05-25

**Authors:** Masami Miki, Takamasa Oono, Nao Fujimori, Takehiro Takaoka, Ken Kawabe, Yoshihiro Miyasaka, Takao Ohtsuka, Daisuke Saito, Masafumi Nakamura, Yasuyuki Ohkawa, Yoshinao Oda, Mikita Suyama, Tetsuhide Ito, Yoshihiro Ogawa

**Affiliations:** ^1^ Department of Medicine and Bioregulatory Science, Graduate School of Medical Sciences Kyushu University Fukuoka Japan; ^2^ Department of Surgery and Oncology, Graduate School of Medical Sciences Kyushu University Fukuoka Japan; ^3^ Division of Bioinformatics, Medical Institute of Bioregulation Kyushu University Fukuoka Japan; ^4^ Division of Transcriptomics Medical Institute of Bioregulation, Kyushu University Fukuoka Japan; ^5^ Department of Anatomical Pathology, Graduate School of Medical Sciences Kyushu University Fukuoka Japan; ^6^ Neuroendocrine Tumor Centre Fukuoka Sanno Hospital, Internal University of Health and Welfare Fukuoka Japan; ^7^ Department of Molecular and Cellular Metabolism, Graduate School of Medical and Dental Sciences Tokyo Medical and Dental University Tokyo Japan; ^8^ CREST Japan Agency for Medical Research and Development Tokyo Japan

**Keywords:** C type lectin domain family 3 member A, lipocalin2, matrix metalloproteinase‐7, pancreatic neuroendocrine tumors

## Abstract

Although the postoperative recurrence rate for pancreatic neuroendocrine tumors (PNETs) is reported to be 13.5%‐30%, the paucity of valuable biomarkers to predict recurrence poses a problem for the early detection of relapse. Hence, this study aimed to identify new biomarkers to predict the recurrence of PNETs. We performed RNA sequencing (RNA‐Seq) on RNA isolated from frozen primary tumors sampled from all localized G1/G2 PNETs resected curatively from 1998 to 2015 in our institution. We calculated differentially expressed genes (DEGs) in tumor with and without recurrence (≥3 years) for the propensity‐matched cohort. Gene ontology analysis for the identified DEGs was also performed. Furthermore, we evaluated the expression levels of candidate genes as recurrence predictors via immunostaining. Comparison of transcriptional levels in tumors with and without recurrence identified 166 DEGs. Up‐ and downregulated genes with high significance in these tumors were mainly related to extracellular organization and cell adhesion, respectively. We observed the top three upregulated genes, C‐type lectin domain family 3 member A (CLEC3A), matrix metalloproteinase‐7 (MMP7), and lipocalin2 (LCN2) immunohistochemically and compared their levels in recurrent and nonrecurrent tumors. Significantly higher recurrence rate was shown in patients with positive expression of CLEC3A (*P* = 0.028), MMP7 (*P* = 0.003), and LCN2 (*P* = 0.040) than that with negative expression. We identified CLEC3A, MMP7, and LCN2 known to be associated with the phosphatidylinositol‐3‐kinase/Akt pathway, as potential novel markers to predict the postoperative recurrence of PNETs.

## 1 INTRODUCTION

The detection of pancreatic neuroendocrine tumors (PNETs) is increasing due to improved diagnostic performance, with the incidence is reported to have increased by 10%.^1^, ^2^

Surgical resection—the only treatment known to achieve a cure—is recommended for locoregional disease control and should still be considered for patients with advanced disease if it can be performed safely.^3^ However, the recurrence rate is reported to be 13.5%‐30%,^4^, ^5^, ^6^, ^7^ with the median duration from resection to recurrence from 2 to 3.3 years.^8^ Certain studies identified mitotic rates,^2^ nonfunctioning status,^2^, ^4^ tumor size > 2 cm,^9^ G2 tumor grade,^4^ N1 status,^9^ and the presence of vascular invasion^4^ as recurrence predictors. Studies have established a risk stratification to predict recurrence by combining TNM stage, Ki‐67,^10^ and functionality.^5^ However, the identification of patients with high risk for recurrence remains challenging.

Currently, transcriptome analysis with microarray and RNA‐sequencing (RNA‐Seq) in PNETs has become widespread, as is also the case with other malignant tumors. Several studies have explored the differences in messenger RNA (mRNA) expression associated with the clinicopathological aspects of PNETs.^11^, ^12^, ^13^, ^14^ For instance, a bioinformatics analysis using GEO datasets identified CXCR4‐CXCL12‐CXCR7 as an overexpressed axis in PNETs with metastasis compared with PNETs without metastasis.^14^

Furthermore, successful transcriptomic analysis to identify PNETs biomarkers has been reported. A PCR‐based 51‐transcript signature (NETest) identified by a multianalyte assay is available as a blood biomarker for PNETs detection and prediction of disease progression.^15^, ^16^, ^17^ The accuracy of this test was superior to that of monoanalyte markers such as chromogranin A and neuron‐specific enolase.

Recently, Kudo et al^18^ identified the downregulation of pancreatic beta cell genes in tumors with simultaneous liver metastasis and found that low expression levels of PAX6, one of the pancreatic beta cell genes, was the most useful predictor for poor prognosis using genome‐wide gene expression analysis.

However, to the best of our knowledge, no study has yet performed comprehensive gene expression analysis to compare tumors with and without recurrence after R0 resection. Herein, we performed RNA‐Seq analysis for PNETs that included those with recurrence. We also confirmed the utility of some DEGs identified as predictive markers using immunohistochemistry.

## 2 MATERIALS AND METHODS

### 2.1 Patients

Candidates for the present study were patients with localized G1/G2 PNETs, as defined by the WHO 2017 classification,^19^ who underwent curative resection between January 1998 and December 2015 and received follow‐up care for three or more years at Kyushu University Hospital. None received neoadjuvant therapy. After surgical treatment for PNETs, all patients underwent laboratory tests and contrast‐enhanced computed tomography (CT) and/or magnetic resonance imaging (MRI) every 3 to 6 months. The disease‐free survival (DFS) was defined as the duration between surgery and detection of a new lesion. For the analysis, we acquired the retrospective clinical information as following: age at surgery, disease stage, tumor size, tumor grade, lymph node status, recurrence. Written informed consent was obtained from each subject as required by the Declaration of Helsinki. All study procedures were approved by an institutional review board (The Human Research Ethics Committee, Kyushu University ID: 29‐183).

### 2.2 Tissue samples

Within 2 hours of resection, tumor and nonneoplastic pancreatic tissues were chopped into 3‐4 mm cubes using a double‐edged razor then placed into the shallow pockets of a freezing plate (Meiko Medical) with 2‐3 mL a small amount of Tissue‐Tek^®^ OCT™ compound (Sakura Finetek) and frozen by liquid nitrogen for 20‐30 seconds until completely frozen as previously described.^20^ We conducted gene expression analysis by performing RNA‐Seq on frozen primary tumor specimens and nonneoplastic pancreatic tissues from the patients.

### 2.3 RNA extraction and sequencing

The tissue‐embedded frozen tablets were transferred into cryovial tubes and stored at −70°C. Frozen sections were then cut into 5‐μm thick specimens according to the general procedure used for conventional OCT blocks, then mounted on glass slides. Hematoxylin and eosin (H&E) staining was performed to confirm that the sample was a neoplastic (or nonneoplastic) section. Then 10 slices of 10‐μm specimen were mixed with RLT solution and the RNA was extracted with RNeasy^®^ Mini Kit (QIAGEN) according to the manufacturer's instructions. RNA‐Seq library preparation using CEL‐Seq2 was performed as previously described,^21^, ^22^ using 10‐30 ng of RNA for each library. The libraries were sequenced on Illumina HiSeq 1500 sequencers (Illumina) and the reads were mapped to the GRCh38 human reference genome assembly using HISAT (v. 2.2.6).^23^

### 2.4 Gene expression analysis

For downstream analysis, the mapped data were converted to counts per million (CPM) and log_2_‐transformed. To obtain enough counts for each gene, samples with at least 1 CPM in the third quartile of the transcripts were extracted as objects. Differential gene expression analysis between representative samples was performed using the R statistical software package, version 3.0.3 (R Project) and edgeR.^24^ Genes with an adjusted false discovery rate (FDR) <0.01 and absolute values of log FC > 1 were considered to be differentially expressed genes (DEGs). Furthermore, to investigate the differences in gene expression patterns between groups, gene ontology enrichment analysis was conducted with the clusterProfiler package in R.^25^

### 2.5 Primary antibodies

We used the following antibodies: rabbit polyclonal CLEC3A antibody (#185282; Abcam) at 1:5000; goat polyclonal LCN2 antibody (AF1757; R&D‐Systems) at 5 μg/mL; rabbit polyclonal MMP7 antibody (10374‐2‐AP; Proteintech) at 1:100; and rabbit polyclonal GHRH antibody (#187512; Abcam) at 1:100.

All antibodies were diluted with phosphate‐buffered saline (PBS).

### 2.6 Immunohistochemistry (IHC)

Four‐micrometer‐thick paraffin tissue sections were deparaffinized with xylene and graded ethanol. After deparaffinization and washing in PBS for three times, antigen retrieval was performed by heating citrate buffer (pH 6.0) at 100°C for 20 minutes. Endogenous peroxidase activity was blocked by incubating the sliced sections with 3% hydrogen peroxide in methanol for 30 minutes. After washing in PBS for three times and incubation in blocking buffer, primary antibody was applied to the tissue sections for 1 hour at room temperature. We applied a streptavidin‐biotin‐peroxidase method for the immunohistochemical reaction against CLEC3A, MMP7, and GHRH using a VECTASTAIN Elite ABC Rabbit IgG Kit (Vector Laboratories). For immunoreaction against LCN2, a polymer system was applied using Goat IgG VisUCyte HRP Polymer (VC004‐025, R&D‐Systems). All tissue sections were visualized with diaminobenzidine (DAB) and counterstained with hematoxylin. Representative photomicrographs were recorded using a digital camera (BZ‐X700; Keyence).

### 2.7 Evaluation of immunostaining

All slides were evaluated by two experienced observers in the absence of patients' clinical data. The immunostaining was assessed in five fields (×200 magnification) for each slide. To evaluate MMP7 expression, the intensity of the staining was graded into four (0 = negative; 1 = weak; 2 = moderate; 3 = strong) and the extent of staining was divided into five categories: 0 = 0%‐5%; 1 = 6%‐25%; 2 = 26%‐50%; 3 = 51%‐75%; and 4 = 76%‐100%. The score was calculated by multiplying the intensity and the extent of staining to produce a range of immunostaining scores from 0 to 12. The immunostaining was considered positive when the scores were ≥3, as a previous study conducted.^26^

For evaluation of LCN2 expression, we applied a four‐grade scoring system^27^ corresponding to the sum of staining intensity (0 = negative; 1 = weak; 2 = moderate; 3 = strong) and the percentage of positive cells (0 = 0%; 1 = 1%‐25%; 2 = 26%‐50%; 3 = 51%‐100% positive cells), as described in a previous study.^28^ A score ≥3 represented a positive expression. We also apply the same method as LCN2 to evaluate the CLEC3A, because there is no literature to refer.

As for GHRH, score was assessed by only staining area; 0 = 0%;1 = 1%‐9%; 2 = 10%‐49%; 3 = 50%‐100%), and a score 3 was considered to be positive expression according to a previous study.^29^

### 2.8 Propensity score matching (PSM)

To compare the expression levels in PNETs with and without recurrence while minimizing the effect of confounders on the selection bias, we calculated propensity scores by using binary logistic regression which included the following variables: gender, age (<60/≥60), tumor grade (G1/G2), functionality (functioning/nonfunctioning), tumor site (Ph/other site), tumor size (<2 cm/≥2 cm), lymph node status (N0/N1), operative approach (open surgery/laparoscopic surgery), history of diabetes mellitus (presence/absence), and BMI (<25/≥25). The resultant score was then used to match tumors with recurrence to those without recurrence for one to one propensity score matching.^30^, ^31^

### 2.9 Statistical analyses

Statistical analyses were performed using JMP ver. 12 (SAS Institute Inc.). The Chi‐squared test or Fisher's exact test was applied to analyze categorical variables. Two‐sided Student's *t*‐tests were used to analyze the differences between continuous values of two independent groups. *P*‐values ≤ 0.05 in the two‐tailed tests were considered significant. Survival curves for relapse were generated using the Kaplan‐Meier method.

## 3 RESULTS

### 3.1 Study population

**Figure 1:**
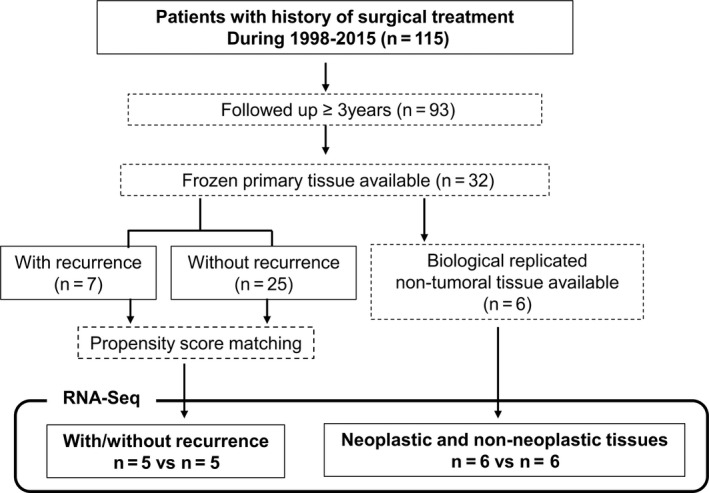
Flowchart for the selection of samples for RNA‐sequencing (RNA‐Seq). Seven and twenty‐five frozen samples of tumors with and without recurrence were available, respectively. The one to one propensity score matching selected five tumor samples in each group (total of 10 samples). We also performed RNA‐Seq for six pairs of samples (neoplastic and nonneoplastic tissue), enabling us to analyze biological replicates

A total of 115 patients underwent R0 surgical resection and were diagnosed with G1/G2 PNETs from 1998 to 2015. Ninety‐three patients received follow‐up care for more than 3 years at our institution. The median follow‐up period for the patients was 83.3 months with a range from 3 to 227 months. In this population, 16 patients (13.9%) experienced recurrence with a median time to recurrence of 19 months and a range from 2 to 77 months; 5 patients (4.3%) died as a result of their PNETs. Twelve in sixteen (75%) patients experienced relapse within 3 years after resection (Figure S1). Figure 1 shows the flowchart for the selection of samples for RNA‐Seq. After excluding 10 samples for RNA‐Seq, 83 samples of the patients followed up more than 3 years were available. Among them, we chose 18 samples (each 9 samples with and without recurrence) for IHC analysis by applying PSM.

### 3.2 DEG analysis and GO analysis of tumor and nonneoplastic tissue

To validate the sequencing data, we conducted pairwise comparisons of differential gene expression profiles by analyzing six independent biological replicates from each tumor and nonneoplastic tissue. We identified 1765 DEGs with false discovery rates <0.01(Table S1). According to GO analysis, upregulated genes in neoplastic tissues were related to transsynaptic signaling and transport function. Downregulated genes in tumors were related to digestion and immune response (Table S2). These results are consistent with a previous study of a microarray‐based gene expression database of PNET tissue samples.^11^

### 3.3 DEG analysis in tumors with and without recurrence

We compared gene expression profiles between tumors with and without recurrence for 10 samples (five pairs) selected by PSM. Table1 shows characteristics of the 10 patients. One hundred and sixty‐six DEGs (114 upregulated genes and 52 downregulated genes) in tumors with recurrence vs without recurrence were identified (Table S3). The significantly upregulated genes in tumors were C*LEC3A*, *MMP7*, *LCN2*, *JCHAIN*,* OLFM4*,* and PIGR*. The significantly downregulated genes were *GHRH*,* PCDH19*, *DAPL1*, *CAPN13*,* ATP6V1B1*, *and FAM132B* (Table 2).

**Table 1:** Characteristics of RNA‐Seq cohort (five cases with recurrence and five cases without recurrence)

Case	Recurrence	Age	Sex	Grade	Ki 67 index	Functioning/NF	Stage	Tumor size (mm)	Tumor site	MEN I	DFS (y)	Observation time (y)	Surgical procedure	Surgical approach	Recurrence site
1	Yes	38	M	G2	16	NF	III	55	Pt	No	0.46		DP	Open	Liver
2	Yes	74	M	G1	1	Glucagonoma	III	40	Ph	No	1.22		PD	Open	Liver
3	Yes	54	F	G2	3	NF	IIB	34	Ph	No	1.60		PD	Open	Lymph node
4	Yes	46	F	G2	5	NF	III	20	Pb	No	1.52		DP	Open	Liver
5	Yes	26	M	G1	1	NF	IIA	45	Ph	No	4.36		PD	Open	Pt
6	No	58	F	G1	1.4	NF	IIA	55	Pt	Yes		11.54	DP	Open	
7	No	65	F	G1	1	NF	IB	28	Ph	No		8.58	PD	Open	
8	No	22	M	G2	5	Insulinoma	IB	26	Ph	No		8.12	PD	Open	
9	No	61	M	G1	2	NF	IA	9	Pt	No		6.61	DP	Laparo	
10	No	54	M	G1	1	NF	IIA	45	Pb	No		4.47	DP	Open	

Abbreviations: DP, distal pancreatectomy; Laparo, laparoscopic surgery; NF, nonfunctioning; Open, open surgery; PD, pancreatoduodenectomy; Pt, pancreatic tail.

**Table 2:** Top 10 up‐ and down‐ differentially expressed genes in PNETs with versus without recurrence

	Gene^a^	Full name	Log FC	Log CPM	*P‐*value	FDR
Up	CLEC3A	C‐type lectin domain family 3 member A	7.449534	6.033011	3.24E−20	4.41E−16
	MMP7	Matrix metallopeptidase 7	6.349328	6.362255	1.45E−19	1.32E−15
	LCN2	Lipocalin 2	6.997872	5.460392	1.39E−17	9.48E−14
	JCHAIN	Joining chain of multimeric IgA and IgM	5.994763	6.460908	1.21E−16	6.60E−13
	OLFM4	Olfactomedin 4	5.480873	5.699948	1.47E−15	6.68E−12
	PIGR	Immunoglobulin (Ig)‐like domain in the polymeric Ig receptor	5.97792	4.957046	6.05E−15	2.36E−11
	GLYATL3	Glycine‐N‐acyltransferase like 3	4.910107	5.993577	3.24E−14	1.10E−10
	PNLIPRP2	Pancreatic lipase related protein 2	5.041008	6.376985	9.56E−14	2.89E−10
	PIP	Prolactin induced protein	5.868028	4.421805	1.32E−12	2.99E−09
	IGLL5	Immunoglobulin lambda like polypeptide 5	4.847139	5.856089	1.46E−12	3.06E−09
Down	GHRH	Growth hormone releasing hormone	−9.53901	7.635353	4.09E−26	1.11E−21
	PCDH19	Protocadherin 19	−5.52537	5.223632	1.41E−13	3.84E−10
	DAPL1	Death associated protein like 1	−5.96133	4.898389	1.31E−12	2.99E−09
	CAPN13	Calpain 13	−5.21838	4.241004	3.81E−10	4.14E−07
	ATP6V1B1	ATPase H + transporting V1 subunit B1	−5.71936	4.048973	3.95E−10	4.14E−07
	FAM132B	Family with sequence similarity 132, member B	−4.78501	4.266354	1.47E−09	1.38E−06
	AMPH	Amphiphysin	−3.4293	6.888431	1.91E−09	1.71E−06
	DCC	DCC netrin 1 receptor	−3.36619	4.150392	1.62E−06	0.00049
	ZCCHC12	Zinc finger CCHC‐type containing 12	−3.33079	4.140206	1.21E−06	0.000385
	NEFM	Neurofilament medium	−3.32982	6.450134	2.64E−08	1.56E−05

Abbreviations: CPM, count per million; Down, downregulated in PNETs with recurrence; FC, fold change; FDR, false discovery rate; Up, upregulated in PNETs with recurrence.

a Genes are arranged in order of FDR from the smallest.

### 3.4 GO analysis in tumors with and without recurrence

As shown in Table 3, upregulated genes in tumors were related to extracellular organization. (eg, extracellular structure organization and extracellular matrix organization) and humoral response (eg, antimicrobial humoral response and humoral immune response). Downregulated genes in tumors were related to cell adhesion (eg, cell‐cell adhesion via plasma‐membrane adhesion molecules and hemophilic cell adhesion via plasmamembrane adhesion molecules).

**Table 3:** Up and downregulated GO terms in gene ontology analysis of significant‐differentially regulated genes in PNETs with versus without recurrence

	ID^a^	GO term (biological process)	Gene ratio	Bg ratio	*P*‐value	*P*‐adjusted
Up	GO:0043062	Extracellular structure organization	42/547	395/17 653	3.11E−12	1.29E−08
	GO:0030198	Extracellular matrix organization	36/547	341/17 653	1.42E−10	2.95E−07
	GO:0019730	Antimicrobial humoral response	19/547	108/17 653	8.02E−10	1.11E−06
	GO:0006959	Humoral immune response	32/547	329/17 653	1.15E−08	1.19E−05
	GO:0006501	C‐terminal protein lipidation	14/547	77/17 653	8.90E−08	7.37E−05
	GO:0007586	Digestion	18/547	134/17 653	1.72E−07	0.000119
	GO:0018410	C‐terminal protein amino acid modification	14/547	86/17 653	3.72E−07	0.00022
	GO:0002526	Acute inflammatory response	22/547	215/17653	9.69E−07	0.000501
	GO:0031214	Biomineral tissue development	16/547	133/17 653	3.66E−06	0.001682
	GO:0001503	Ossification	29/547	371/17 653	5.16E−06	0.002135
	GO:0061844	Antimicrobial humoral immune response mediated by antimicrobial peptide	10/547	59/17 653	1.20E−05	0.004533
	GO:0001649	Osteoblast differentiation	19/547	205/17 653	2.19E−05	0.007549
Down	GO:0098742	Cell‐cell adhesion via plasma‐membrane adhesion molecules	23/411	242/17 653	1.24E−08	4.16E−05
	GO:0007156	Homophilic cell adhesion via plasma membrane adhesion molecules	18/411	158/17 653	3.04E−08	5.11E−05

Note

Genes expressed with the difference of the log FC value >2 or <−2 were defined as GO analysis objects.

Abbreviations: down, downregulated In PNETs with recurrence; Up, upregulated In PNETs with recurrence.

a GO are listed in order of *P*‐adjusted from the smallest.

### 3.5 IHC analysis of CLEC3A, MMP7, LCN2, and GHRH expression

**Figure 2:**
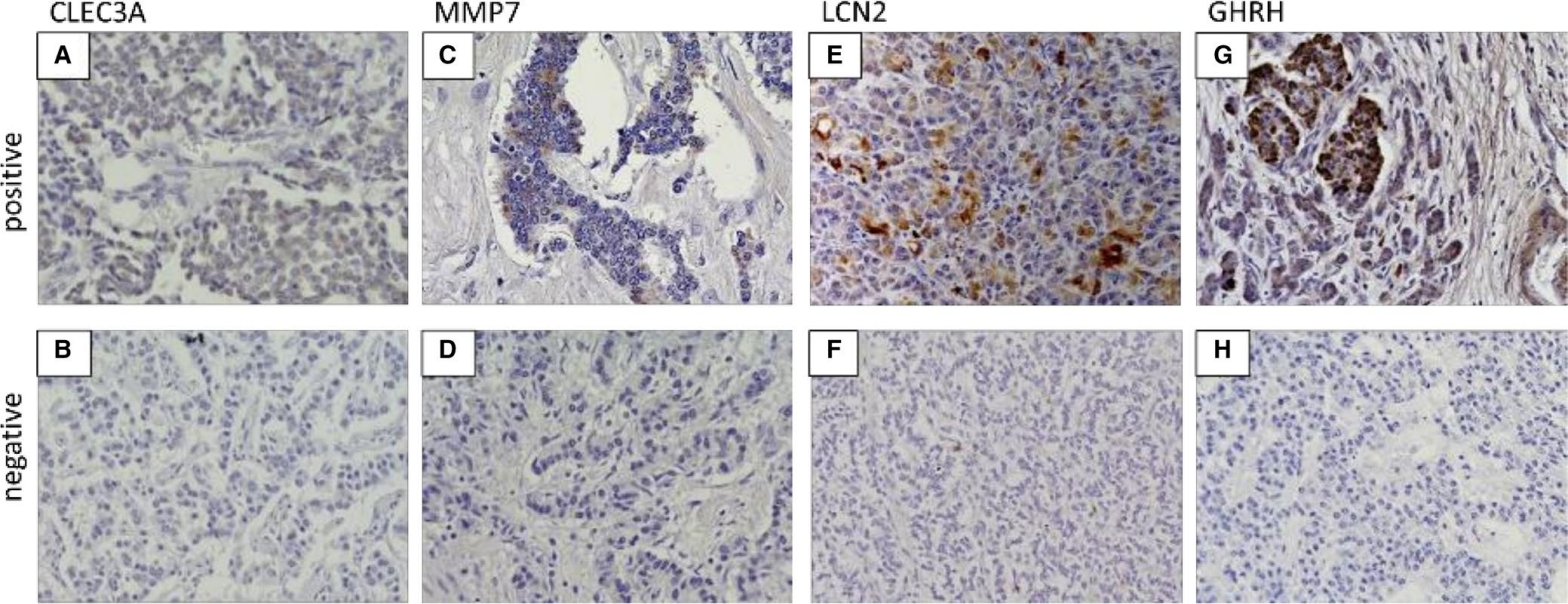
Representative immunohistochemical staining patterns of formalin‐fixed, paraffin‐embedded (FFPE) sections (magnification 200×) for CLEC3A (A, B), MMP7 (C, D), LCN2 (E, F), and GHRH (G, H). Upper images (A, C, E, G) are positive patterns. Lower images (B, D, F, H) are negative patterns

We chose a few genes—upregulated *CLEC3A*, *LCN2*, and *MMP7* and downregulated *GHRH*—in cases with relapse as candidates for recurrence predictors. To confirm the protein expression and location of these genes, we conducted immunostaining for each tumor sample with and without recurrence. Table 4 shows the clinical characteristics of the IHC cohort (nine samples in each) before and after PSM. The confounding factors became balanced between the two groups. Figure 2 displays representative immunohistochemical staining patterns of formalin‐fixed, paraffin‐embedded sections for CLEC3A, MMP7, LCN2, and GHRH. The immunostaining positivity for each gene and clinical characteristics of 18 patients are summarized in Table 5.

**Table 4:** Patient background and clinical characteristics of IHC cohort pre‐ and post‐adjustment with one‐to‐one nearest‐neighbor matching method

	Pre‐propensity score matching (n = 83)			Post‐propensity score matching (n = 18)		
	With recurrence (n = 11)	Without recurrence (n = 72)	*P*‐value	With recurrence (n = 9)	Without recurrence (n = 9)	*P*‐value
Gender—male	3 (27.3)	30 (41.6)	0.51	5 (55.6)	6 (66.7)	0.62
Age (years old, median [range])	56 [28‐82]	55.5 [19‐79]	0.83	53 [33‐74]	54 [22‐64]	0.66
Functionality—Functioning	2 (18.2)	23 (31.9)	0.49	2 (22.2)	2 (22.2)	1.00
Tumor size (mm, median [range])	30 [5‐120]	15 [5‐57]	0.002*	7 (77.8)	6 (66.7)	0.60
Presence of LN metastasis	5 (45.5)	6 (18.3)	0.005*	5 (55.6)	4 (44.4)	0.63
Differentiation (G1/G2)‐G2	9 (81.8)	10 (13.9)	<0.0001*	2 (22.2)	3 (33.3)	0.60
Tumor Location (Ph/Pb/Pt)‐Ph	7 (62.6)	45 (62.5)	0.94	5 (55.6)	5 (55.6)	1.00
Approach (Open/Laparo)‐Open	10 (90.1)	38 (52.8)	0.002*	8 (88.9)	8 (88.9)	1.00
Association with MEN1—yes	3 (27.3)	7 (9.7)	0.10	2 (22.2)	3 (33.3)	0.59
History of DM—yes	2 (18.2)	10 (13.9)	0.70	2 (22.2)	2 (22.2)	1.00
BMI (median [range])	21.2 [17.6‐34.2]	22.5 [16.9‐31.9]	0.13	22 [17.6‐26.9]	21.5 [18.1‐26.3]	0.72

Note

Numerical value represents mean number (%) or number [range]. *P‐*value was calculated using Chi‐squared test or Whitney's *U* test.

Abbreviations: F, functioning; Laparo, laparoscopic surgery; NF, nonfunctioning; Open, open surgery; Pb, pancreatic body; Ph, pancreatic head; Pt, pancreatic tail.

* *P* < 0.05, significant.

**Table 5:** IHC results and characteristics of 18 patients

Patient No.	IHC Expression								Clinical features											Duration (y)	
	CLEC3A		MMP7		LCN2		GHRH		Age	Sex	Grade	Ki‐67 index	Tumor size (mm)	Location	Functionality	MEN I	LN metastasis	Recurrence site	Stage	DFS	Observation period
	Score^a^	•/×^b^	Score	•/×	Score	•/×	Score	•/×													
R1	0	×	3	•	0	×	1	×	33	M	G1	0.1	30	Pt	Nonfunctioning	Yes	(+)	Liver	IIB	3.8	10.3
R2	4	•	6	•	4	•	3	•	74	M	G1	1	40	Ph	Glucagonoma	No	(+)	Liver	III	1.2	5.9
R3	4	•	4	•	3	•	2	×	55	F	G1	1	5	Ph	Gastrinoma	Yes	(+)	Liver	IIB	6.5	10.5
R4	4	•	2	×	4	•	3	•	40	M	G2	2.6	25	Pb	Nonfunctioning	No	(−)	Liver	IB	6.8	19.5
R5	4	•	4	•	2	×	3	•	54	F	G2	3	34	Ph	Nonfunctioning	No	(+)	Lymph node	IIB	1.6	6.5
R6	4	•	9	•	4	•	2	×	51	F	G2	8	15	Pb	Nonfunctioning	No	(−)	Liver	IA	2.6	6.5
R7	12	•	4	•	0	×	3	•	61	F	G2	9.85	27	Ph	Nonfunctioning	No	(−)	Liver	IIA	0.2	5.8
R8	2	×	2	×	4	•	2	×	66	M	G2	12	37	Ph	Nonfunctioning	No	(+)	Liver	IV	1.0	4.6
R9	4	•	0	×	4	•	0	×	38	M	G2	16	55	Pt	Nonfunctioning	No	(+)	Liver	IV	0.5	3.8
N1	0	×	0	×	2	×	2	×	58	F	G1	1.4	55	Pb	Nonfunctioning	Yes	(−)	‐	IIA	11.4	11.5
N2	0	×	0	×	0	×	3	••	51	M	G1	2	5	Pt	NF + Gastrinoma	Yes	(+)	‐	IIB	4.7	4.7
N3	1	×	0	×	0	×	1	×	54	F	G2	3	9	Pb	Gastrinoma	No	(−)	‐	IA	9.0	9.0
N4	0	×	0	×	0	×	0	×	58	M	G2	3	45	Ph	Nonfunctioning	No	(+)	‐	IIB	5.8	6.0
N5	0	×	1	×	4	•	3	•	53	M	G2	3	57	Ph	Nonfunctioning	No	(+)	‐	IV	4.8	5.2
N6	0	×	0	×	0	×	0	×	64	M	G2	4	43	Ph	Nonfunctioning	No	(+)	‐	III	3.5	3.7
N7	0	×	0	×	0	×	0	×	34	M	G2	4.2	33	Pb	Nonfunctioning	No	(−)	‐	IB	13.1	13.1
N8	0	×	0	×	0	×	3	•	22	M	G2	4.8	26	Ph	Insulinoma	No	(−)	‐	IB	3.6	3.6
N9	0	×	0	×	0	×	2	×	47	F	G2	7	16	Pt	Nonfunctioning	Yes	(−)	‐	IA	3.1	3.1

Abbreviations: DFS, disease‐free survival; N1‐9, cases without recurrence; Pb, pancreatic body; Ph, pancreatic head; Pt, pancreatic tail; R1‐9, cases with recurrence.

a Scores were determined as described in the method.

b •, positive (score ≥ 3); ×, negative (others).

### 3.6 Disease‐free survival in patients with CLEC3A, MMP7, LCN2, and GHRH

**Figure 3:**
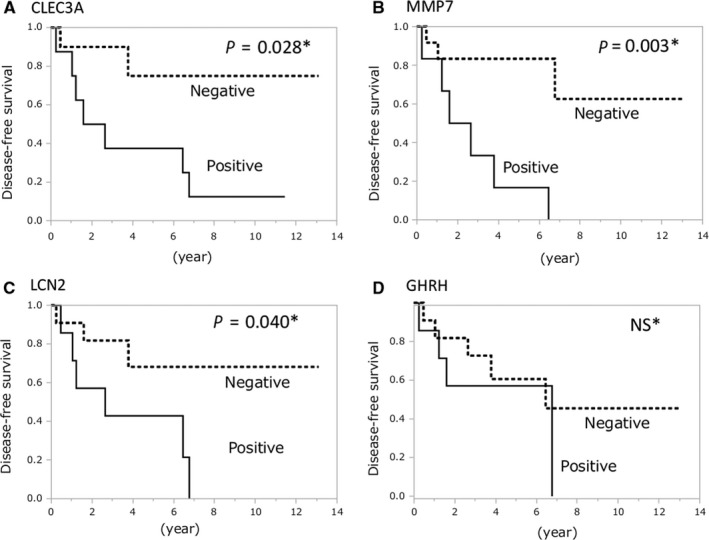
Disease‐free survival after the resection in patients. Significant differences were observed between patients with CLEC3A (A), MMP7 (B), LCN2 (C), and GHRH (D) by log‐rank test

Kaplan‐Meier survival analysis showed that recurrence rate in patients with positive expression of CLEC3A (*P* = 0.028), MMP7 (*P* = 0.003), and LCN2 (*P* = 0.040) was significantly higher than patients with negative expression by log‐rank test. Recurrence curves were shown in Figure 3. No recurrence occurred among patients with negative expression for all three genes in the resected tumor. In contrast, no significant difference was observed in recurrence rate between patients with positive and negative GHRH expression.

## 4 DISCUSSION

Though PNETs with postoperative recurrence are not rare, no biological predictive markers have yet been established. We compared gene expression levels in PNETs with recurrence and to those without recurrence using RNA‐seq, aiming to identify important biological factors and predictive markers for recurrence.

The major finding in the present study is that the top three upregulated genes *CLEC3A*, *LCN2*, and *MMP7* showed significantly higher expression at the protein level in tumors with recurrence than in those without recurrence by IHC, which might be useful for predicting recurrence.

Although significant downregulated genes in tumor with recurrence were related to cell‐cell adhesion as mentioned in the result, the difference in gene expression between recurrence and nonrecurrence was too small to expect to be a marker predicting recurrences. Meanwhile, the most downregulated gene, GHRH displayed the prominent expression difference. Furthermore, GHRH has been well known to be secreted by neuroendocrine tumors ectopically.^32^, ^33^ Considering the possibility that GHRH is a valuable marker with high accuracy, we investigated GHRH protein expression in tumors. However, no relationship between GHRH expression and postoperative recurrence was observed.

Additionally, we selected the top three upregulated genes, *CLEC3A*,* MMP7*, and *LCN2*, because the expression of CLEC3A, MMP7, and LCN2 have been reported to be useful markers in other kind of malignant tumors, and known to be associated with the PI3K (phosphatidylinositol 3‐kinase)‐Akt pathway, which plays an important role in a subset of PNETs 34 as described below.

CLEC3A, belonging to the superfamily of C‐type lectins, has been reported to be expressed in cartilage and is associated with osteoarthritis.^35^ CLEC3A expression has also been reported in breast cancer tissue and high CLEC3A expression significantly correlated with poor prognosis. A previous study indicated that CLEC3A promotes invasion and metastasis of breast cancer cells by activating the PI3K/Akt signaling pathway.^36^

Matrix metalloproteinases are a family of zinc‐dependent endopeptidases.^37^ MMP7 is secreted specifically by epithelial cells^38^ and its overexpression has been observed in many tumor types including colorectal cancer,^38^ bladder cancer,^39^ gastric cancers,^40^ pancreatic cancer,^41^ and esophageal cancer.^42^ Additionally, a study of prostate cancer with neuroendocrine differentiation demonstrated that MMP7 leads to increased endothelial cell coverage and enlarged vessel size, resulting in increased invasion of neighboring tissue.^43^ It has been reported that upregulation of MMP7 expression is caused by activation of the PI3K/Akt pathway and is required for the migration and invasion of colorectal cancer cells.^44^ A study of gastric cancer indicated that the EGF‐induced overexpression of MMP7 depends on the PI3K signaling cascade.^45^

Moreover, a previous study revealed that MMP7 cleaves CLEC3A bound to the cell surfaces of various cancer cell lines, including colon, breast, and lung cancer cells.^46^ The cleavage of CLEC3A by MMP7 promotes the migration of cancer cells thorough the suppression of cell‐adhesion activity.^46^

LCN2, a 25 kDa secreted glycoprotein^47^also known as neutrophil gelatinase associated lipocalin (NGAL), was originally isolated with matrix metalloproteinase 9 in human neutrophils and identified as an antibacterial factor of natural immunity. Thereafter, it was found to be expressed in various cells and to participate in the growth, development, and differentiation of many tissues.^48^, ^49^ Currently, the roles of LCN2 in human neoplasia attract attention because its increased expression has been observed in malignant tumors^48^, ^49^ and its pro‐tumoral effects have been observed in epithelial ovarian cancer,^28^ pancreatic cancer,^50^ and esophageal cancer.^47^ In colon cancer, it has also observed that LCN2 overexpressed during the transition from adenoma to carcinoma, indicating that LCN2 drives tumor progression.^51^, ^52^ Meanwhile, the antitumoral and antimetastatic effects of LCN2 were demonstrated in hepatocellular carcinoma,^53^ lung cancer,^54^ and pancreatic cancer.^55^ The cell type‐specific function of LCN2 remains controversial. According to previous studies, the role of LCN2 in cancer metastasis might be related to plakophilin3 (PKP3) loss, which leads to an increase in invasion, tumor formation, and metastasis.^56^, ^57^, ^58^

A microarray analysis showed that the expression levels of MMP7 and LCN2 increased upon PKP3 loss in multiple cell lines.^59^ LCN2 and MMP7 may play a crucial role in cell invasion and tumor formation upon PKP loss via the PI3K/Akt‐dependent pathway.^60^

**Figure 4:**
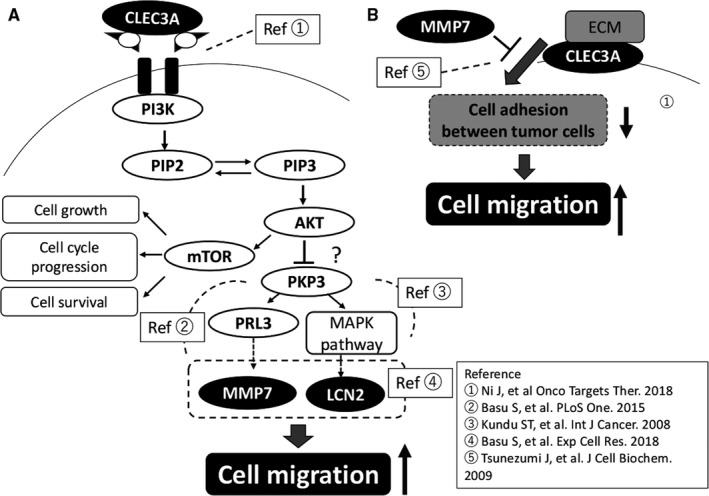
The conceivable migration pathway related to PNETs based on literatures. Ref., reference; Ref.1, Ni J, et al Onco Targets Ther. 2018; Ref.2, Basu S, et al. PLoS One. 2015; Ref.3, Kundu ST, et al. Int J Cancer. 2008; Ref.4, Basu S, et al. Exp Cell Res. 2018; Ref.5, Tsunezumi J, et al. J Cell Biochem. 2009. CLEC3A, C type lectin domain family 3 member A; ECM, extracellular matrix; LCN2, lipocalin2; MMP7, matrix metalloproteinase‐7; mTOR, mammalian target of rapamycin; PI3K, phosphoinositide 3‐kinase; PIP2, phosphatidylinositol‐4,5‐bisphosphate; PIP3, phosphatidylinositol‐3,4,5‐trisphosphate; PKP3, Plakophilin3; PRL3, phosphatases of regenerating liver 3

Referring to the previous studies, CLEC3A, MMP7, and LCN2 could play roles in tumor cell migration through the pathway as shown in Figure 4. Briefly, PI3K/AKT signaling pathway, activated by CLEC3A^36^ and other molecules, upregulates MMP7^60^ and LCN2^59^ thorough loss of PKP3, which promotes cell migration^56^ (Figure 4A). CLEC3A is also located in cell membrane, and support adhesion between tumor cells by interacting with Extracellular matrix (ECM) on the cell surface.^46^ However, MMP7 promotes cell migration by cleaving CLEC3A, which inhibits the function of CLEC3A (Figure 4B).

To the best of our knowledge, this study is the first to show that these three genes may play some roles in the recurrence of PNETs. Further studies are required to elucidate the detailed mechanism.

The study has some limitations. First, we were unable to analyze the relationship between gene expression and survival time because the mortality rate in all patients in the study was less than 4%. Second, the results of expression analysis using bulk RNA samples are likely influenced not only by tumor cells but also by the tumor stroma. Third, we evaluated the immunopositivity by one‐slice staining. A tissue microarray immunostaining analysis would be preferable for a more accurate evaluation. Moreover, investigation should have been confirmed by using a validation cohort setting. The adequate samples, however, were not available. Further prospective evaluation in our institution is under planning.

In conclusion, we demonstrated that the recurrence potential in PNETs is characterized by the upregulation of genes related to ECM organization and cell adhesion. This study identified three genes—*CLEC3*, *MMP7*, *and LCN2*—that contribute to the prediction of postoperative recurrence of PNETs in clinical practice.

## CONFLICT OF INTEREST

The authors of this article declared they have no conflicts of interest.
