# Supplementary figures and images for: CLEC3A, MMP7, and LCN2 as novel markers for predicting recurrence in resected G1 and G2 pancreatic neuroendocrine tumors
Source: Cancer Med. 2019 May 25;8(8):3748–60. doi: 10.1002/cam4.2232 (PMC6639196; doi:10.1002/cam4.2232)

**Figure S1**

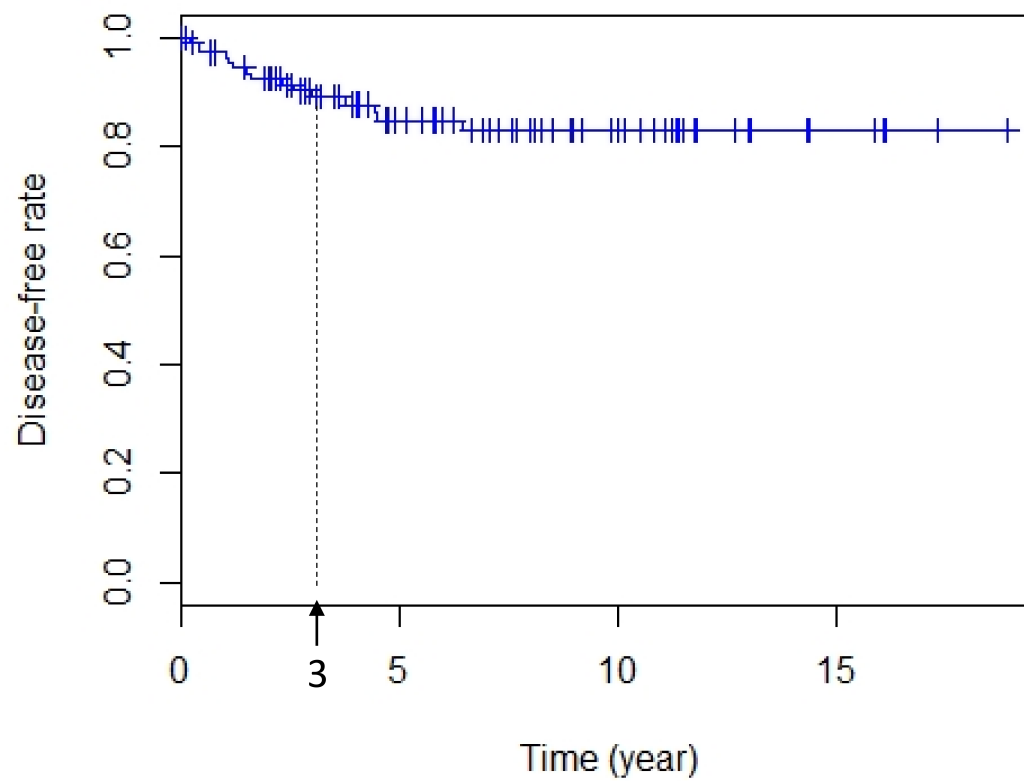

Supplement: Supplementary file 1 [file CAM4-8-3748-s001.pdf]
